# Scaling up an Evidence-Based Exercise Intervention for Wide-Scale Implementation: A Conceptual Roadmap Using the PRACTIS Framework

**DOI:** 10.3390/healthcare14060720

**Published:** 2026-03-11

**Authors:** Louise Declerck, Trinh L. T. Huynh, Robert W. Motl

**Affiliations:** 1 Exercise Neuroscience Research Lab, Department of Kinesiology and Nutrition, Applied Health Sciences, University of Illinois Chicago, 1919 W Taylor St, 545 AHSB (MC517), Chicago, IL 60612, USA; 2 Department of Medicine, University of Illinois Chicago, 840 S Wood St, Suite 1020N, Chicago, IL 60612, USA

**Keywords:** exercise, multiple sclerosis, implementation and dissemination science

## Abstract

Background: Exercise is safe and beneficial for managing symptoms, preventing progression, and enhancing the physical and mental well-being of people with multiple sclerosis (MS). Such evidence has supported prescriptive guidelines delivered alongside behavioral interventions to promote and sustain exercise behavior. Evidence-based exercise training interventions now exist for people with MS, such as Project GEMS, a home-based program grounded in MS-specific exercise training guidelines and supported by principles of behavior change based on social–cognitive theory. The field is now ready for the process of translating evidence-based programs from research settings into clinical or community contexts for greater reach and accessibility, but dissemination and implementation remain significant challenges. Objectives: The current paper presents a conceptual roadmap for implementing a validated home-based exercise training intervention for people with MS, originally tested in controlled research environments, within the broader community context. This is guided by the PRACTIS framework for scaling up physical activity/exercise interventions. Roadmap: Our paper presents a conceptual example along with details regarding strategic adaptations for tailoring the remote GEMS program for a diverse and wide MS population, integrating input from community stakeholders and ensuring long-term sustainability through collaborative researcher–stakeholder partnerships. Such an initiative may bridge the gap between research and practice in the domains of exercise training and behavioral interventions for people with MS and further serve as a scalable model for promoting exercise behavior in other neurological conditions by expanding accessibility for effective digital resources. Conclusion: The design of the exercise intervention discussed in this paper offers one example and conceptual pathway for expanding access for evidence-based exercise programs at the wider level.

## 1. Introduction (Background and Rationale)

Beliefs about physical activity and exercise for people with multiple sclerosis (MS) have undergone significant changes over the last century [1]. Initially, exercise was thought to be dangerous or potentially harmful for MS, and people with the disease were advised to avoid physical exertion [2]. This caution stemmed largely from the observation of Uhthoff’s phenomenon, as it was believed exercise-induced heat could temporarily worsen neurological symptoms, such as blurred vision [3]. Additionally, there were concerns that exertion could exacerbate underlying neurological deficits, leading to increased fatigue and relapses [4,5]. This negative view of physical activity and exercise changed in the late 20th century as scientific research, including the first randomized controlled trials (RCTs) on the topic, challenged prevailing views and demonstrated the safety and benefits of exercise for MS [6,7,8,9]. Since then, a substantial body of research, including meta-analyses, has demonstrated that physical exercise improves the physical and mental well-being of people with MS, alleviates symptoms such as depression and fatigue, and enhances cognitive function and overall quality of life [10,11,12]. Some research suggests that physical activity may exert disease-modifying effects, potentially reducing lesions and relapse rates and slowing disability progression [13,14]. This evolving evidence base culminated in the development of exercise guidelines for MS in 2013 [15] that were later refined in 2020 by the National MS Society through clinical and research experts’ opinion-based recommendations [16]. Together, it is recommended that all adults with mild to moderate MS engage in 30 min of moderate-intensity aerobic activity 2–3 days per week and strength training for major muscle groups 2–3 days per week [15,16]. For those with greater disability levels, experts recommend engaging in 10–15 min of aerobic activity, as well as upper- and lower-limb range of motion exercises 3 or more days per week [16]. Those guidelines are further complemented by evidence on the safety of exercise in MS, as summarized in the literature [17].

The evidence of benefits and safety is countered by research consistently demonstrating that people with MS engage in low levels of physical activity and physical exercise. For example, a recent systematic review with a meta-analysis on physical activity levels reported a statistically significant difference, of moderate magnitude, in physical activity levels between people with MS and healthy controls [18]. Both light physical activity and moderate to intense physical activity are reduced in MS [19,20,21]. Additionally, another recent meta-analysis indicated significantly higher levels of sedentary behaviors in those with MS than healthy controls [19].

The low levels of physical activity and exercise displayed by the MS population are indicative of a research to practice gap. Indeed, the efficacious interventions developed in research environments fail to reach the broader community, and this may be due to a combination of individual, organizational, and systemic barriers [22,23]. Research programs often provide structured, controlled, and expert-led environments, whereas community settings may lack the necessary resources and expertise to implement these interventions effectively and sustainably. Many of the exercise interventions tested among people with MS through RCTs have not undergone systematic development with the explicit end-goal of community implementation [24], which considerably decreases the potential to be scaled up or translated into long-term, community-based practices. That disconnect underscores a significant public health challenge, as the majority of people with MS remain unable to access or benefit from evidence-based exercise programs that could improve health and quality of life. This collectively underscores a critical need to focus on interventions that demonstrate strong potential to overcome the “implementation cliff” and successfully serve the broader MS community.

**Figure 1:**
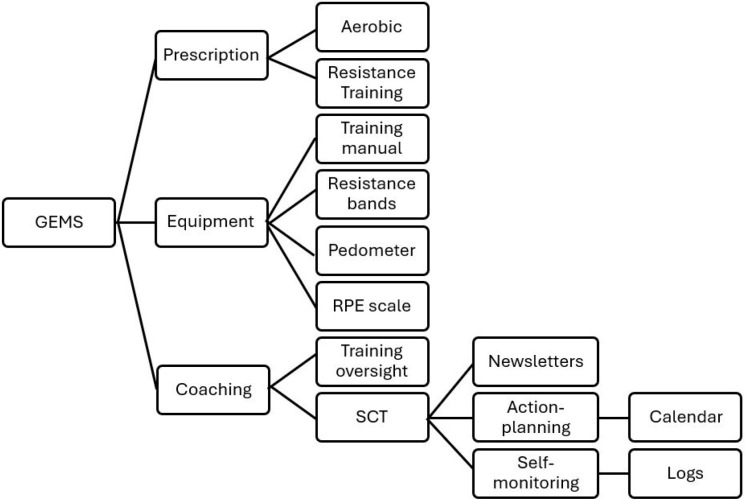
Outline of the Guideline for Exercise in Multiple Sclerosis (GEMS) program as provided previously in a different protocol paper [26]. RPE = rate of perceived exertion; SCT = social–cognitive theory.

We have developed and established a robust and pragmatic approach for remote delivery, support, and monitoring of aerobic and resistance exercise training based on prescriptive guidelines for persons with MS (i.e., the Guidelines for Exercise in MS [GEMS] program) [25,26]. This approach was based on 15 years of deliberate research and included the development of a platform for remote delivery of aerobic and resistance exercise training consisting of an aerobic and resistance exercise prescription, appropriate exercise equipment, one-on-one coaching, action planning via calendars, logs for self-monitoring, and SCT-based newsletters. The program and its components are described in Figure 1.

The GEMS program has demonstrated promising feasibility, efficacy, and effectiveness across diverse populations with MS, including tailored adaptations for Black individuals with MS [26,27,28], older adults with MS [29], Hispanic individuals with MS [30], and wheelchair users with MS [31], indicating improvements in exercise behavior and health-related qualitiy of life [32]. Notably, the GEMS program delivered remotely has been non-inferior for improving walking performance compared with in-person, supervised delivery of the same program [33]. This consistent evidence, along with the remote nature and low-resource delivery model, highlights the potential of the GEMS program as an example of a scalable intervention for promoting exercise behavior and managing symptoms in the broader MS community.

**Figure 2:**
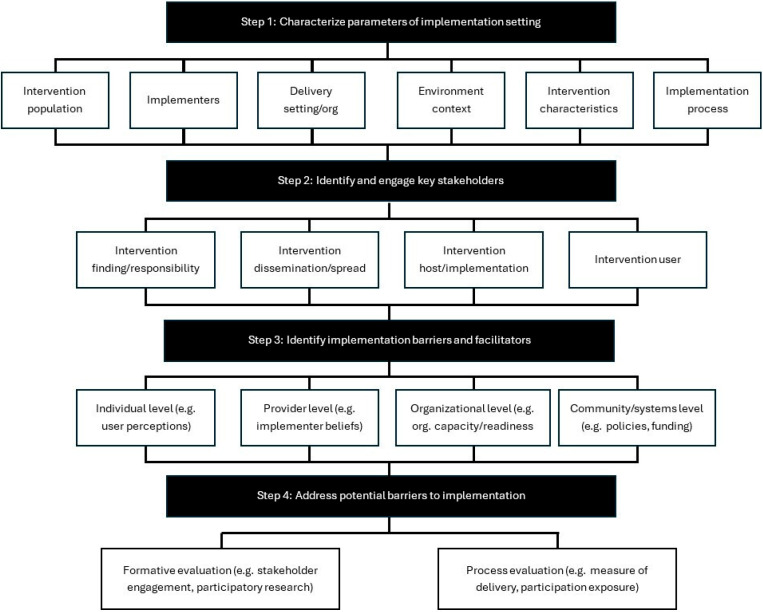
PRACTIS guide framework [34].

The PRACTical planning for Implementation and Scale-up (PRACTIS) framework is a strategic guide designed to help bridge the gap between research-based physical activity interventions and real-world application [34]. This framework highlights early and thoughtful planning to ensure that interventions are feasible, sustainable, and impactful and delivered effectively in diverse settings when scaled across diverse clinical and community environments. The framework includes four iterative steps: understanding the implementation setting, engaging key stakeholders, identifying barriers and facilitators, and proactively addressing those barriers (Figure 2). Each step encourages collaboration, adaptability, and foresight, making it a practical tool for anyone aiming to move from pilot projects to widespread adoption. Applying PRACTIS to scale up the GEMS program is a logical next step and represents a strong, practical example for describing the process. The GEMS program—a patient-centered, remote, and community-driven initiative that focuses on empowering people with MS to increase consistent and sustainable exercise behavior—would be an ideal candidate as an example for applying the PRACTIS structured approach to scale-up. The framework could help anticipate and navigate real-world challenges, such as resource limitations, stakeholder buy-in, and contextual differences across settings. By using the PRACTIS framework, the GEMS program, or any other program, we can maintain fidelity to its core mission while adapting to the unique needs of each community it enters. This ensures that scale-up efforts are inclusive, evidence-informed, and built for long-term success.

The GEMS program is an established, evidence-based intervention that has been developed and evaluated through several RCTs, and it is ripe for translation into clinical and/or community settings as a critical and practical next step in the cascade of intervention development and implementation. Indeed, this evidence-based approach has great potential to be implemented into real-world care settings and provide people with MS a health-behavior-based approach for managing symptoms, improving quality of life, and perhaps managing MS and its progression. This paper provides a conceptual adaptation pathway for translating the GEMS program into a fully automated version (GEMS-Auto). In this conceptual, proposed model, all components (i.e., exercise prescriptions and SCT-based behavior change components) would be delivered remotely via simulations of the delivery of intervention components without requiring in-person coaching involvement. The core components of GEMS-Auto would be converted into automated videos that simulate instructions and interactions between a coach and a participant, utilizing an online delivery, coaching-free intervention format [35]. This modification of the original GEMS program, delivered in research settings, targets the sustained implementation of the program by people with MS in the community. Importantly, GEMS-Auto is not yet developed, nor is it implementation ready; instead, this paper aims to present a roadmap for its future development. To that end, this paper articulates a conceptual adaptation pathway for translation of the evidence-based GEMS intervention, outlining key considerations and planned processes required to successfully translate GEMS into a highly implementable GEMS-Auto. Using the PRACTIS framework as a theoretical guide, this paper identifies stakeholder-informed and context-specific factors necessary to support future scale-up and wide-scale implementation. Implications and future directions are further presented.

## 2. Step-by-Step Implementation Process Guided by the PRACTIS Framework

The translation of GEMS into GEMS-Auto, as a conceptual example, would involve the four steps of the PRACTIS guidelines, as summarized in Table 1.

### 2.1. Step 1: Characterize Implementation Setting Parameters

The translation of the GEMS program into the GEMS-Auto might begin with identifying the intervention population and implementation setting parameters. The implementation setting is defined as the physical, social, and cultural context/environment where the physical activity intervention is implemented, as well as who, what, how, and all underpinned contexts for decision making and strategies for implementation planning and testing [34]. The GEMS-Auto program would be ambulatory-based, which aims to be delivered to adults with MS who have mild to moderate disability and are able to walk independently or with unilateral support. We opted for this MS subpopulation as previous studies indicate that people with MS who have mild to moderate disability account for about 60–70% of people with MS in the US [36]. This aims to broaden the reach of this intervention within the limits of safety for participants in an unsupervised physical activity/exercise program. Other inclusion criteria include (1) adults with MS (18 years of age or older), (2) no relapses or exacerbations within the past 30 days, (3) insufficiently active based on the physical activity guidelines for people with MS [25], (4) having email and Internet access, (5) having necessary technical literacy to use computers/smart devices effectively for the e-learning approach, and (6) willing to participate and complete the intervention.

Participants could be recruited through clinician referral through MS clinics, as previous research indicates that people with MS prefer receiving exercise promotion initially through healthcare providers who are trusted and considered a credible source for people with MS [37]. To streamline referral to GEMS-Auto and implement it into routine clinical practice without increasing provider burden, healthcare providers such as neurologists might be instructed to introduce GEMS-Auto during standard MS care visits. This would be facilitated by providing neurologists with an operational blueprint that would further serve as referral support. This document would provide them with clear information on the intervention (i.e., what their patient will be getting) and include a brief three-step process: first, ensure the patient is eligible for the program in terms of presenting with mild to moderate disability; second, guide participants through the six questions of the Physical Activity Readiness Questionnaire [38] and determine whether they have any exercise contraindication that may compromise safe delivery of GEMS-Auto; and third, deliver a structured and scripted screening process consisting of three questions: (1) Do you currently engage in regular aerobic and resistance exercise? (2) Are you interested in starting or increasing your participation in an exercise program? and (3) May I provide you with a referral to a remotely delivered exercise program? Patients who answer positively and indicate interest would be provided with a GEMS-Auto pamphlet during the visit. This pamphlet would describe the benefits of exercise in MS and evidence on the positive findings of the original GEMS program in the research settings applied across MS sub-populations. It could further describe what to expect when participating in the study. These physical pamphlets would be provided to clinicians for distribution to potential participants to promote the intervention. In addition, ongoing support would be provided to clinicians for any questions or concerns and to ensure they have sufficient copies of the pamphlets to distribute to new patients.

The GEMS intervention might be updated to GEMS-Auto to ensure this online package is entirely coaching-free and self-directed, which can facilitate its delivery through clinical settings via referrals from neurologists or healthcare providers. This would prepare the intervention for sustainability in lower-resource settings where online materials can be distributed or accessed remotely, and participants can self-direct their use of these guides for behavior change and engaging in physical activity behaviors. The core components of the GEMS program would be maintained in GEMS-Auto, including aerobic exercise prescription, resistance exercise prescription, and the SCT-based behavior change component. Those three core components would be delivered through a program manual, exercise equipment package, and SCT-based newsletters supported by videos. The videos would be organized into different modules that mirror the structure and progression of the original GEMS intervention and align with the stages outlined in the physical program materials (i.e., exercise manual and logbook). They would be brief (≤5 min) and depict scripted video simulations of a behavior coach interacting with a person with MS. The videos would model the delivery of the aerobic and resistance exercise prescriptions, explain the exercise and the intensity, demonstrate exercise adaptations, review safety procedures, and provide guidance on the use of the logbook and the calendar. The videos could also provide answers to frequently asked questions. The videos would be embedded within and throughout the physical program materials (i.e., exercise manual and logbook) and accessed via clearly marked QR codes and direct web links to a secured web-based platform consisting of sequential, stage-based modules. The online material would complement the exercise manual and logbook, and progression of the videos would be contingent upon completing prior videos to preserve intervention sequencing. Each participant would receive a unique, secure login, enabling individual-level tracking of website platform access, video views, module initiation and completion, and frequency and duration of engagement. To further support adherence and ensure fidelity to the program, participants with no recorded website activity for a two-week period might automatically receive standardized reminder emails prompting re-engagement or offering assistance, thereby maintaining program exposure while preserving the fully automated design. Together, this would allow for monitoring of their adherence to the online GEMS-Auto module. Core online engagement metrics might include the number of logins, time spent on the platform per session and in total throughout the program, number of modules completed, video access frequency, and percentage of full-length video completion. This would be complemented by tracking exercise adherence, compliance, and potential adaptations through the provided logbooks (consistent with the original GEMS program) to determine fidelity across all intervention components.

The exercise prescription was based on the current physical activity guidelines for adults with MS. The guidelines specify 30+ min of moderate to vigorous intensity aerobic exercise (2–3 times per week) and resistance training targeting major muscle groups (2–3 times per week) [16]. These guidelines are for people with MS who have mild to moderate disability (i.e., classified by either an examiner-established Expanded Disability Status Scale (EDSS) score 0–6.5 [14,15] or a Patient-Determined Disease Steps (PDDS) score of 0–6 [39]). However, for the fully automated GEMS, eligibility might be more conservatively restricted to a target MS population with EDSS 0–5.5 (PDDS scores 0–4), indicating sufficient ambulatory status to safely engage in the walking-based exercise prescription, to decrease risk concerns with a more impaired sample undertaking a home-based program without direct supervision of exercise sessions.

Aerobic exercise prescription: The aerobic training focuses on walking, the most common mode of exercise for people with MS [40], which can be administered and monitored in non-supervised settings and aims to build up to 30 min of moderate-intensity walking (i.e., a pace of 100 steps per minute) three times per week. The step rate of 100 steps per minute corresponds with moderate-intensity exercise in adults with MS and would be monitored by a provided pedometer throughout the program, as detailed in the previous study [25]. For the first two weeks, all participants would begin with an accommodation period, during which they would walk briskly at 100 steps per minute for 10 min three times per week. This 2-week period would serve as a benchmark for selecting a trajectory for the remaining 14 weeks of the intervention. From weeks 3 to 16, the participant would gradually progress their walking duration toward 30+ min using three trajectories (i.e., Orange, Blue, and Green) for individualization. The aerobic exercise training guideline for the Orange, Blue, and Green trajectories would be achieved by 6, 8, and 10 weeks, respectively. Participants would be instructed to walk as fast as is safely possible, even if they cannot achieve the prescribed intensity, and to complete the aerobic portion of the intervention at home and/or in the community (e.g., park, shopping mall, high school track). This would be explained in the manual, along with a QR code for accessing video simulations.

Resistance exercise prescription: The strength training would consist of 1–2 sets with 10–15 repetitions of 5–10 exercises targeting the lower body, upper body, and core muscle groups performed three times per week using resistance bands [25]. The lower body exercises are the chair raise, calf raise, knee flexion, knee extension, and the lunge; the upper body resistance exercises are the shoulder row, shoulder raise, elbow flexion, and elbow extension; and the core exercise is the abdominal curl. The manual would further include exercise modifications to ensure the safety of participants. For the first two weeks, all participants would begin with one set of 10 repetitions of five exercises, including the chair squat, shoulder row, elbow flexion, abdominal sit-up, and calf raise in each session. During this period, exercise intensity would be standardized using the lightest resistance band (204 lbs). This first, 2-week period could be deemed an accommodation period and serve as a benchmark for selecting a trajectory for the remaining 14 weeks of the program. From weeks 3 to 16, participants would progress gradually based on three trajectories (i.e., Orange, Blue, and Green) to meet the minimum dose of resistance exercise on weeks 6, 8, and 10, respectively. During this period, participants could self-select resistance bands based on the amount of resistance that permits 10–15 repetitions with appropriate technique. All participants would undertake the resistance program with the provided elastic bands at home and be provided with equipment, including a training manual and an RPE scale, to monitor exercise intensity. This could be explained in the manual, along with a QR code for accessing video simulations.

SCT-based behavior change component: The original GEMS program involves one-on-one semi-structured coaching sessions with an MS exercise specialist (i.e., a behavioral coach) and six newsletters, which were delivered throughout the program. The frequency of the coaching chats and newsletters is displayed in Table 2. These sessions focus on exercise training guidance and oversight, discussion of the behavioral strategies of action planning and self-monitoring using logbooks and calendars, providing feedback and information on appropriate exercise techniques and strategies, and discussion of newsletters; these latter parts are based on SCT and designed for optimizing exercise compliance. During the coaching chats, participants and the behavioral coach discuss the levels of difficulty of the exercise intervention and how to progress toward meeting the exercise guidelines. These core elements of the behavior change component of GEMS would be translated into eight online video modules simulating the one-on-one, semi-structured sessions to discuss action planning, self-monitoring strategies, and newsletter content (based on SCT-based topics, namely, outcome expectations, self-monitoring, goal-setting, self-efficacy, barriers, and facilitators) and to address frequently asked questions. These could be accessible to the study participants by scanning a QR code (or typing the URL) highlighted in the received exercise training manual.

We could focus on reach, equity of access for our participants, and retention in the implementation process from the very beginning through multiple-prong approaches [41]. To reach potential participants with MS, we would create partnerships with MS clinics and clinicians to form a collaborative care model, along with support from community liaisons across the country [42]. Partnership with MS clinics and clinicians provides access to clinical settings, which is the backbone of the implementation process and aligns with our mission of promoting physical activity and exercise as a complementary approach for MS care. This might further spread awareness about the benefits of physical activity, promote interventions, and serve as a credible source to deliver the intervention. To ensure the reach of the program and equity of access for disadvantaged subgroups, the intervention materials, including recruitment materials, would be culturally tailored to the diverse pool of participants, supported by the collaborative care model with clinicians and community partners [42]. Additionally, the effort of translating the GEMS program into a technology-based GEMS-Auto intervention using the Internet delivery platform, pre-recording video simulations, and setting up a QR code for mobile app access increases potential reach to diverse populations, given that mobile use among people with MS is prevalent [43]. Retention of the intervention would be supported by offering flexible support from the study team [44].

### 2.2. Step 2: Identify and Engage Key Stakeholders

The key stakeholders in the development and implementation of GEMS-Auto include people with MS, healthcare providers (the ENRL team), clinicians, and community partners. People with MS could be the driving force of the intervention design and recipients of the implementation process. It is crucial that the intervention and its implementation meet the wants and needs of the participants. In the provider role, the ENRL team is responsible for intervention design and adaptations, remote support, and intervention monitoring and oversight. We would serve as the primary intervention host and implementation body, while intervention users would be people with MS. Clinicians play a vital role in our collaborative care model to deliver the GEMS-Auto intervention, as they bridge the intervention from research settings to clinical settings and refer the intervention to potential participants. MS clinics and centers could also serve as primary delivery sites for the GEMS-Auto intervention in the future stages of implementation. In particular, the automated components of GEMS-Auto could be integrated into patient-support programs; and this further step of embedding GEMS-Auto within the clinical setting would enhance the reach and adoption of the intervention by leveraging existing clinical infrastructure and provider support. Additional stakeholders include community organizations (e.g., the National MS Society, iConquerMS, the Multiple Sclerosis Association of America) as funding agencies and partners in supporting and advocating for the implementation of the GEMS-Auto intervention.

### 2.3. Step 3: Identify Contextual Barriers and Facilitators

The primary objective of Step 3 of the PRACTIS guide is to identify contextual barriers and facilitators that may influence the successful implementation and scale-up of interventions in real-world practice settings [34]. These influences operate across individual, provider, organizational, and community/system levels. Accordingly, we systematically identified potential barriers and facilitators relevant to successful implementation and scale-up of the fully GEMS-Auto program across MS care and community settings. At the individual level, potential barriers related to user characteristics encompass severity and fluctuation of MS symptoms (such as fatigue, heat sensitivity, physical impairments, pain, etc.), disability progression [45,46], and low exercise self-efficacy [45,47]. In addition, perceptions of the intervention’s effectiveness and its ability to address unmet needs relative to existing options may greatly influence uptake by people with MS [34]. However, there is substantial evidence indicating that people with MS are aware of the benefits of exercise [48,49], and this suggests that perceived efficacy and relevance are unlikely to represent major barriers and more likely to represent facilitators in the context of delivering GEMS-Auto. Finally, perceived motivation to engage in exercise may play a key role in either impeding or facilitating long-term engagement with the full GEMS-Auto program [50]. This may be positively or negatively affected by the extent to which participants enjoy the intervention [51,52].

Given the nature of GEMS-Auto, provider-level barriers related to implementer characteristics such as knowledge, education, and skill [34] might be minimal. Indeed, the translation of one-on-one coaching to online modules with similar social–cognitive theory-based content allows for high-fidelity delivery of the behavior change without depending on provider-specific resources, such as time, knowledge, education, skill, attitude, etc. [34,35]. This is highly relevant, as healthcare providers within neurorehabilitation largely do not provide behavior change in the clinic due to a lack of confidence or understanding of how to apply behavior change strategies [53,54]. It is essential to note, however, that the successful implementation and uptake of the GEMS-Auto program would largely depend on MS healthcare providers referring MS patients to the program. Potential barriers to this referral process may include limited provider awareness of GEMS, negative beliefs about the safety of remotely delivered exercise programs, time constraints during clinical encounters and competing clinical priorities, and uncertainty about the program’s suitability for individual patients. To support safe and efficient referral, GEMS-Auto would incorporate clear eligibility criteria, screening guidance and scripts, and a contraindication checklist, which would be provided to neurologists expressing interest in promoting the program to their patients. Participants would not be granted access to GEMS-Auto unless they confirm that they were specifically referred to the program by a licensed healthcare provider. Adverse event monitoring and escalation pathways would need to be explicitly defined at the start of the program. Participants would then receive standardized instructions on how to recognize, react, document, and report adverse events related to exercise, with clear escalation procedures for events requiring clinical follow-up. These would be tiered as follows: (1) automated self-management guidance for mild, transient symptoms; (2) automated recommendation to contact a healthcare provider for persistent or moderate symptoms; and (3) explicit instruction to seek urgent or emergency care (with emergency contact information displayed) for severe symptoms, such as chest pain, acute neurological change, or loss of consciousness. Clear instructions would allow participants to differentiate between expected post-exercise soreness and warning signs suggestive of relapse or medical concern. Healthcare providers referring patients to GEMS-Auto might also be informed of these monitoring procedures, thereby reinforcing shared understanding of safety responsibilities. Referral barriers may be further mitigated by providing good-quality evidence regarding the safety and efficiency of the program to care providers (see step 4) and engaging allied clinicians in the development, dissemination, and implementation process (see step 2). Moreover, a qualitative study highlighted the interest of healthcare providers in the promotion of exercise within MS care, together with the need for resources that providers can easily offer to patients [37]. Additional facilitators may include the provider’s personal attributes (e.g., their own experience with exercise) and their knowledge regarding the benefits of exercise in MS.

Similarly to the provider level, barriers occurring at the organizational level and stemming from setting characteristics are reduced by the remote delivery of the GEMS-Auto program. Such characteristics include work climate, administrative support, and organizational climate, amongst others [34]. The cost of setting and delivering the intervention [55] needs to be considered, as it may be key to ensuring wide-scale implementation of the GEMS-Auto program. The anticipated cost of delivering the intervention would be low and only include the cost of equipment (manual, logbook, resistance bands, and pedometer), but this cost could still pose a barrier for some individuals, limiting the accessibility and equity of the intervention’s reach. Furthermore, accessing the online modules requires some basic level of digital literacy, and this may prove to be a barrier to a minor part of the MS population.

Finally, barriers at the community/system level (e.g., politics, policy, funding, etc.) would be considered in the GEMS-Auto program [34]. The GEMS-Auto program would ideally be promoted, hosted, and funded through and by large MS advocacy groups that would provide potential users (referred to the program by MS healthcare providers) with the necessary equipment, though this largely depends on the capacity, readiness, and network of advocacy groups. Key facilitators include the global recognition and visibility of MS advocacy groups as leaders in advancing MS research and care, their strong engagement with patients, and their extensive collaborations with other MS advocacy organizations, which collectively serve to unify the field and strengthen coordinated efforts to support individuals with MS.

### 2.4. Step 4: Address/Assess Barriers to Effective Implementation

The purpose of the fourth and last step is to ultimately address the barriers identified in step 3 by refining the intervention and the implementation process, and this requires formative evaluation and process/outcome evaluation [34]. In addition, the existing implementation literature on effective strategies for implementation and dissemination can guide this step of the PRACTIS framework. For example, Mun et al. recently emphasized the importance of creating high work engagement, or one’s positive attitude and motivation towards work, among healthcare providers, as this significantly predicts implementation of evidence-based practices [56]. This opens a potential avenue for addressing provider-level barriers through improvements in the overall organizational culture. Moreover, a systematic review on facilitators, barriers, and considerations for the implementation of healthcare innovation lists several themes and sub-themes of considerations for successfully implementing new interventions, and these include facilitating training and sharing information supporting innovation effectiveness [57].

In the specific context of the GEMS-Auto program, participatory research with stakeholders identified in step 2 would enable feedback and subsequent targeted refinement of the intervention’s processes and materials, including the novel video modules simulating behavioral coaching. This would enhance the user-friendliness of the program, as well as its relevance to the target population, and, in turn, potentially improve its sustainability. In addition, focus groups with potential users of the GEMS-Auto program could address the aerobic and resistance components of the intervention, as this may serve to maximize both efficacy and enjoyment of the exercise program. It should be noted, however, that previous users of the original GEMS program reported high levels of satisfaction with both the intervention itself and the materials used in the intervention [32]. We therefore believe that changes proposed by such a formative evaluation would be minimal. Creating an advisory group of MS healthcare providers, including neurologists, would allow for clear communication regarding the safety and efficacy of GEMS-Auto and create an opportunity for discussing issues, concerns, and ideas for improvement providers may have. Furthermore, providers would be given the space to share experiences regarding the utility and revisions of the operational blueprint and referral process to promote the GEMS-Auto program among their MS patients, which could allow for the formulation of best practices and ensure implementation fidelity. At the community/system level, ongoing monthly to yearly consultations with MS advocacy groups, who would ultimately host and fund the intervention on a long-term basis, are needed to further strategize and optimize scaling-up of the program [58].

Process/outcome evaluation would be assessed as well, and this can be done through feasibility and pilot trials or other studies using various methodologies that aim to understand the effectiveness and uptake of the GEMS-Auto program [34]. Such outcomes can be evaluated in parallel to intervention testing [34], which warrants the use of hybrid effectiveness–implementation trials [59]. As a vital first step, the hybrid effectiveness–implementation trial might compare GEMS-Auto to usual care, as this comparison group truly reflects current clinical practices and their effects. In that case, the inferential target would be superiority, and this would need to be taken into account for a power analysis. The more specific articulation of this study design and methodology, together with important considerations related to the statistical plan (including how to address missing data) and mitigation of different sources of bias (including confounding due to disease progression), is warranted and might be developed further in a future protocol paper. Altogether, it is important that process/outcome evaluation be based on carefully selected single or multiple theories, models, and frameworks [60]. An appropriate candidate framework is the RE-AIM by Glasgow, Vogt and Boles [61]. This model evaluates the overall public health impact of multilevel health-promoting interventions across five domains of interest, namely, reach, efficacy, adoption, implementation, and maintenance [61]. Its scope, together with its popularity, far-reaching applicability, and ability to evolve to address emerging issues [62], are well-suited to conduct a comprehensive process evaluation of the GEMS-Auto program. Such an evaluation could be guided by the specific outcomes of interest within each of the five domains listed in Table 3, below.

## 3. Discussion

GEMS-Auto has the potential to be a pragmatic and scientifically strong response to persistently low PA and exercise participation in the MS population by maintaining the core evidence-based components of the original GEMS program [32] while removing resource-intensive elements (i.e., the one-on-one coaching with a highly trained behavioral coach) that limit implementation at a large scale. The use of the PRACTIS framework within this work has allowed for a better understanding of the implementation setting and the range of stakeholders [34]. Importantly, this analysis highlighted the importance of leveraging trusted referral pathways through MS clinics and healthcare providers [63], as this would enhance credibility and, in turn, uptake among people with MS [64]. Embedding referral to GEMS-Auto within existing clinical workflows has the unique potential to normalize exercise as a core component of MS care and aligns with what and how neurologists would like to promote exercise to MS patients [37]. In addition, we emphasized the critical role of developing strong partnerships with advocacy organizations, as they are the ultimate funders of the low-cost GEMS-Auto. Together, this model supports system-level reach and sustainability, positioning GEMS-Auto as a viable public health strategy rather than a time-limited research intervention.

Another strength of our structured analysis of GEMS-Auto is the identification of facilitators and/or barriers and how to overcome the latter [34]. These stem from user (e.g., MS-related characteristics), provider (e.g., time constraints and competing clinical priorities), organizational (cost and technology), and community/system (e.g., advocacy groups’ capacity) levels and might be addressed through formative and outcome evaluation. Monitoring these barriers as they occur in real-life situations, developing solutions, and evaluating their effects on the overall implementation of GEMS-Auto would be critical as the program rolls out. In particular, qualitative research could play a central role in refining the intervention through personal feedback. Recruiting the first cohort of GEMS-Auto participants at the end of the four-month intervention period for in-depth interviews or focus groups would provide valuable insight into user experiences, perceived benefits, barriers to engagement, and unmet needs. These data could inform iterative improvements to intervention materials, delivery format, and implementation strategies, thereby strengthening long-term sustainability. In general, adopting an iterative approach might be essential for continuously improving the fit of the program with the broad community it intends to serve.

This paper focuses on implementation planning, and subsequent rigorous evaluation of GEMS-Auto is essential to support broader dissemination. A logical next step is a randomized controlled trial designed to evaluate the effectiveness of GEMS-Auto in relation to exercise behavior, fitness, and health-related outcomes in people with MS. Such a trial would provide critical evidence regarding whether automated delivery retains the behavioral and clinical benefits observed in earlier, coach-led versions of GEMS. In line with implementation science best practices, a hybrid effectiveness–implementation design [59,65] would allow for simultaneous assessment of outcomes such as reach, adoption, fidelity, and maintenance [62].

If successful, subsequent equity of access to GEMS-Auto might be a critical consideration for further scaling GEMS-Auto [42]. Although the remote delivery model enhances reach, disparities may persist due to differences in digital literacy, language proficiency, and access to resources. Future iterations of GEMS-Auto might explore modifications to printed and digital materials to improve readability, accessibility for individuals with varying levels of digital and health literacy, and cultural relevance. Indeed, adaptations to support participants with low digital and health literacy may include simplifying the online platform to one linear pathway with a single clear “Next” button and minimal or one-click login requirements. Program content could use short sentences, common words, and active voice, supplemented with icons, diagrams, and simple illustrations, alongside access to human technical support to assist with navigation and troubleshooting. In addition, for those without reliable internet or device access, exercise manuals may be enhanced to include behavioral change content provided in the videos, though future work may be needed to determine whether this remains equally effective. Regarding cultural relevance, qualitative research already provides information on how to tailor GEMS, its components, and its material to the African-American MS population [27]. Language accessibility represents another important avenue for expanding reach. Developing versions of GEMS-Auto in languages other than English, such as Spanish [30], could substantially increase equity in access given the diversity of the MS population [66]. Ideally, such adaptations would go beyond direct translation and include cultural tailoring of examples, imagery, and messaging to ensure relevance and engagement. This could include modifying the video and manual content by having a presenter/narrator of a similar race and ethnicity as the target population [27]. Partnerships with people with MS of these different cultural and linguistic groups could be essential to guide these adaptations and facilitate dissemination within diverse communities.

Finally, although GEMS-Auto is specifically tailored to adults with MS and grounded in MS-specific exercise guidelines [15,16], the underlying intervention structure may be applicable to other chronic neurological or long-term health conditions, such as Parkinson’s disease. Indeed, core determinants of physical activity behavior, such as self-efficacy, outcome expectations, perceived barriers, and self-regulation, upon which the original GEMS program was built are shared across populations [67,68]. We therefore believe the program detailed in this paper, and the implementation insights it provides, could be applied beyond MS to benefit others, as well. In the future, evidence from other populations with neurological or chronic conditions may help inform which components of GEMS-Auto are broadly transferable and which require population-specific modification.

Several limitations, or threats to the wide-scale implementation of GEMS-Auto, should be acknowledged. First, the original GEMS program is designed for adults with MS who have mild to moderate disability [25,32] and currently excludes those with more severe physical impairments, although testing this approach in wheelchair users is underway [31]. If the data collected in that study supports GEMS as safe, feasible, and effective among people with MS with more severe disability, future adaptations of GEMS-Auto might take this into account in order to extend the implementation of the fully automated approach to the broader MS community. However, thus far, the feasibility and safety of home-based exercise training have not been the object of research within the severely disabled sub-sample of the population, which warrants the current limited eligibility criteria [16]. In addition, GEMS-Auto requires sufficient digital access and literacy, which may limit generalizability to individuals with more advanced cognitive impairments or limited access to technology. More extensive work is needed to explore avenues for efficiently modifying all components of GEMS-Auto, including the exercise program itself, so that it is accessible to people with greater impairment. Second, while automation reduces resource burden and enhances scalability, the absence of real-time human interaction with a behavioral coach may affect adherence to the exercise program for some participants who particularly rely on interpersonal support. This highlights the need to undertake an effectiveness–implementation trial to test adherence to GEMS-Auto and identify its correlates. Ideally, this data would serve clinicians to identify potential non-adherents to the approach who might then be referred to a different, non-automated route. Finally, the GEMS exercise program is based on MS exercise guidelines published in 2013 and 2020 [15,16], and these undergo updates to recommended exercise prescriptions. In that case, GEMS-Auto would be adapted to reflect this evidence-based change.

## 4. Conclusions

GEMS-Auto holds great potential for an important and innovative advancement in the translation of exercise science into real-world MS care. By applying the PRACTIS framework, this work demonstrates a structured, proactive, and actionable approach to planning for implementation and scale-up and for addressing barriers that could limit the impact of the benefits of the automated approach for people with MS. The automated, low-resource, and theory-driven design of GEMS-Auto would offer a promising pathway for expanding access to an evidence-based exercise program at the wider community level. Future research and adaptations focusing on effectiveness, implementation outcomes, and equity will be critical to realizing the full public health potential of this intervention.

## Figures and Tables

**Table 1:** GEMS-Auto operationalization based on the PRACTIS guide.

	Automated Home-Based Physical Activity/Exercise Behavioral Intervention for People with MS (GEMS-Auto)
Target outcome Population Setting	PA, exercise Adults with MS (18+ years), EDSS ≤ 5.5 or PDDS ≤ 4; having Internet and email access Clinical setting Home-based/community-based
Step 1: Characterize implementation setting parameters	**Place**: Healthcare clinics with home-based online components Online platform where the intervention material would be stored and distributed **People** and **Process**: Participants referred from MS centers to receive the 4-month intervention The intervention would be a fully automated, self-directed, home-based exercise program involving 3 days/week of 30+ min of aerobic exercise (i.e., moderate-intensity walking) and 3 days per week of resistance exercise (i.e., 1–2 sets, 10–15 repetitions of 5–10 movements targeting the major muscle groups) **Provisions**: Program manual, exercise prescriptions, and video simulations for SCT-based behavioral coaching sessions and exercise instructions, educational newsletters, exercise equipment (i.e., yoga mat, resistance bands, pedometer), activity log, calendar, and incentives **Principles**: Intervention—home-based intervention based on SCT and behavioral strategies (i.e., action planning, self-monitoring, goal setting, reviewing behavioral goals, and feedback) to increase physical activity and improve MS health outcomes Implementation—Used an integrated research–practice partnership within an existing care delivery system to increase system-wide scale-up
Step 2: Identify and engage key stakeholders	Stakeholders represent people with MS, providers/coordinators (ENRL team) who are responsible for intervention design and adaptation, remote support, and implementation oversight, clinicians, MS clinics/centers, and NMSS as the funding agency and supporting/advocating for implementation of the intervention
Step 3: Identify contextual barriers and facilitators	User level: (i) MS-related characteristics (e.g., disability level, symptom severity, symptom fluctuation), (ii) exercise self-efficacy, (iii) perceptions of intervention effectiveness and fit for unaddressed need, (iv) motivation, (v) enjoyment Provider level: (i) awareness and knowledge, (ii) beliefs about safety, (iii) time constraints and competing clinical priorities, (iv) fit with patient demographic, (v) personal experience with exercise Organizational level: (i) cost, (ii) technology Community/system level: (i) partner advocacy group’s capacity, readiness, and network, (ii) visibility and role of advocacy groups
Step 4: Address/assess barriers	Formative evaluation: participatory research with stakeholders, focus groups with potential users, advisory group of MS healthcare providers, consultations with advocacy groups Process/outcome evaluation: hybrid effectiveness–implementation, RE-AIM

**Table 2:** Behavioral change session content.

Week 1 Introduction to program	Video 1: Clarification of materials received and initial questions; explanation of program; planning exercise schedule; using the logbook and Qualtrics; Newsletter 1; exercise expectations; exercise outcomes; importance of this knowledge.
Week 2 Outcome expectations	Video 2: Compliance with program; using the manual, logbook, and Qualtrics. Identifying personal outcomes.
Week 3 Choosing a program	Video 3: Compliance with program; comparison of Orange, Blue, and Green programs; choosing a program; Newsletter 2; self-monitoring defined; benefits of self-monitoring; importance of this knowledge.
Week 4 Self-monitoring	Video 4: Compliance with program; using your pedometer; understanding exercise intensity.
Week 5 Goal-setting	Video 5: Compliance with program; setting SMAART goals; performing resistance training exercises correctly; tracking progress; Newsletter 3; specific, measurable, adjustable, action-oriented, realistic, and time-limited exercise-related goals defined; importance of this knowledge.
Week 7 Self-efficacy	Video 6: Finding your self-confidence; what to do when you feel like quitting; involving family; Newsletter 4; self-efficacy defined; experiencing success, choosing role models, accepting encouragement, and managing physical and emotional responses; reminder that the program is specific to persons with MS.
Week 11 Overcoming barriers	Video 7: Identifying your barriers; making plans to overcome obstacles; dealing with MS symptoms; Newsletter 5; exercise barriers defined; common barriers (facilities, social, and symptoms); strategies to overcome barriers.
Week 15 Identifying facilitators	Video 8: How to keep going on your own; making adjustments as needed; setting future goals; Newsletter 6; exercise facilitators defined; common facilitators (having a goal, enjoyment, social support, knowledge); using facilitators long-term.

**Table 3:** RE-AIM framework and outcome measures.

RE-AIM Domain	Definition	Example of Specific Outcome Measures to Evaluate in the Automated GEMS Program
Reach	Number, proportion, and representativeness of the target population who are willing to participate in the intervention	Number of participants at baseline and number of dropouts Demographic and MS-related characteristics of the participants
Efficacy	Success rate of the intervention or its impact on subjective or objective outcomes of interest	Participation in leisure-time exercise Adverse events MS symptoms and disability level; this could combine both patient-reported outcome measures (e.g., fatigue severity scale) and objective measures of physical function (e.g., isometric strength assessment, cardiopulmonary exercise test)
Adoption	Number, proportion, and representativeness of settings that adopt the intervention	Number and proportion of neurologists who refer their patients to the GEMS-Auto program Demographic characteristics of the neurologists
Implementation	Extent to which the intervention is implemented as intended in the real world; includes adaptations to the intervention and costs of implementation; at the user level, includes use of the intervention and implementation strategies	Cost of delivering the GEMS-Auto program Adherence and compliance to the different components (i.e., aerobic and resistance training, SCT-based videos) of the GEMS-Auto program; this could combine both online engagement metrics (e.g., number of videos watched until the end) and logbook data regarding exercise session completion
Maintenance	Extent to which behavior is sustained after the intervention and the program becomes part of routine organizational practices and policies	Number and proportion of neurologists who refer their patients to the GEMS-Auto program after 6 months Physical activity levels of participants at 4 months post-intervention follow-up [29]; this could combine both self-reported exercise behavior (e.g., Godin Leisure Time Exercise Questionnaire) and objective measures of physical activity (e.g., accelerometry)

Refs. [24,61,62].

## Data Availability

No new data were created or analyzed in this study.
